# The gap between perceived mental health needs and actual service utilization in Australian adolescents

**DOI:** 10.1038/s41598-022-09352-0

**Published:** 2022-03-31

**Authors:** Md Irteja Islam, Fakir Md Yunus, Samia Naz Isha, Enamul Kabir, Rasheda Khanam, Alexandra Martiniuk

**Affiliations:** 1 Faculty of Medicine and Health, School of Public Health, The University of Sydney, Sydney, NSW Australia; 2 Centre for Health Research and School of Business, The University of Southern Queensland, Toowoomba, QLD Australia; 3 Centre for International Public Health and Environmental Research, Bangladesh (CIPHER,B), Dhaka, Bangladesh; 4 Department of Psychology and Neuroscience, Faculty of Science, Dalhousie University, Halifax, Canada; 5 CAPABLE-A Cambridge-Led Programme in Bangladesh (In Affiliation With ICDDR,B and IEDCR), University of Cambridge, Cambridge, UK; 6 Centre for Health Research and School of Sciences, The University of Southern Queensland, Toowoomba, QLD Australia; 7 Office of the Chief Scientist, The George Institute for Global Health, Level 5/1 King Street, Newtown, NSW 2042 Australia; 8 Dalla Lana School of Public Health, The University of Toronto, 155 College St Room 500, Toronto, ON M5T 3M7 Canada

**Keywords:** Health care, Health services, Medical research, Epidemiology, ADHD, Anxiety, Depression, Psychiatric disorders, Psychosis, Comorbidities, Risk factors

## Abstract

Despite being highly prevalent, adolescent mental health problems are undertreated. To better understand the mental health treatment gap, we assessed the prevalence and correlates of help-seeking, including perceived need for care and access to that care. Data were drawn from Young Minds Matter (YMM) survey—the second Australian child and adolescents survey of mental health and wellbeing. Parent-reported data and self-reported child data were combined into one dataset to analyse 2464 Australian adolescents aged 13–17 years. We employed bivariate and multivariate logistic regression models to assess the correlation between independent variables (professionally assessed with mental disorders only, self-reported self-harm/suicidality only and both) and their distribution over outcome variables (perceived need and service use). Mental disorders include depression, anxiety, ADHD and conduct disorder. Our study revealed 15.0%, 4.6% and 7.7% had professionally assessed with mental disorders only, self-reported self-harm/suicidality only and both, respectively. Overall, 47.4% and 27.5% of adolescents respectively perceived need for care and used services in the past-12-months. While among those only who perceived the need, only 53% of adolescents used any services. Professionally assessed with mental disorders only, self-reported self-harm/suicidality only and both were associated with higher likelihood of perceived need and service use (*p* < 0.001 for all). However, adolescents who self-reported self-harm/suicidality only were not found to be significantly associated with service use among those who perceived the need for care. Adolescents who perceived the need for mental health care but did not seek care represent a treatment gap. Our results suggest the importance of reducing the wide treatment gap that exists between need and care.

## Introduction

Adolescent mental health is a global concern^1,2^. It is estimated that six out of the top ten causes of disease burden among adolescents are attributable to mental and substance use disorders^3–5^. According to the United Nations recently published report on children’s mental health, globally, more than 13% of adolescents aged between 10 and 19 years are reported to have a diagnosed mental disorder^6,7^. About 40% of these diagnosed mental disorders were attributable to anxiety and depression, followed by attention-deficit-hyperactivity-disorder (ADHD), conduct disorder, autism and substance use disorder^6,8,9^. Moreover, self-reported mental health issues such as self-harm and suicidality are also omnipresent in adolescents—differing in intensity and duration, affecting quality-of-life and have long-term detrimental effect^10–12^. Studies^13,14^ reported that the prevalence of self-harm and suicidality are strikingly high among 13–17-year-olds adolescents and are strongly associated with suicide—the fourth most common cause of death in teenagers worldwide^15,16^. Previous studies also suggest that due to the major demographic, environmental, socioeconomic, and political changes in recent times, the burden of poor mental health is rising among adolescents^17–20^. Exacerbating this, COVID-19 pandemic-associated restrictions (e.g., social distancing, quarantine, lockdown, school closure) imposed worldwide have negatively impacted adolescent mental health in an unprecedented manner^19,21–23^. Further, it is estimated in 2021 that the invisible economic cost due to mental health problems in adolescents is approximately $387 billion per year^7^.

In Australia, like in other developed nations, the high prevalence of adolescent mental health problems has remained over the past decades^5,24^. According to the most recent statistics in Australia, 1 in every seven 4–17-year-olds (about 14 percent) has been diagnosed with one or more mental disorders in the previous 12 months^10,25^. The highest percentages of children and adolescents in Australia were diagnosed with ADHD, which is around 7.4%, followed by anxiety (6.9%), depression (2.8%) and conduct disorder (2.1%)^26,27^. Moreover, ADHD, depression, anxiety and conduct disorder are altogether accountable for 12% of the global burden of disease (GBD) in Australia^11,25^. Further, these mental disorders have been identified as risk factors for self-harm and suicidality among adolescents^28–30^. In Australia, the most recently estimated prevalence of self-harm and suicidal ideation in nationally representative studies of adolescents was 8 and 7.5%, respectively^28,29^. As published by the Australian Bureau of Statistics (ABS), these statistics have not altered much over the previous years, making suicide the top cause of death in Australians under 25 years^31,32^. Additionally, it has been reported that Australian adolescents are currently experiencing even higher rates of mental health problems (e.g., stress, depression, anxiety, health risk behaviours) due to the COVID-19 pandemic^22,23,33^.

Recently, it has been reported that although a significant proportion of adolescents worldwide live with mental health problems, service access is limited^7^. Evidence-based mental health services can help alleviate this burden; however, studies reveal that even in the developed nations, parents and adolescents often underuse potentially accessible mental health resources^4,5,34^. In Australia, a significant proportion of adolescents who need to access services for diagnosed mental disorders or self-reported mental health issues do not receive it adequately, either due to lack of mental health literacy among adolescents or parents, social stigma and/or due to lack of resources^26,27,35–38^. When those services are needed and could help, not using services that exist is a significant public health concern^39^. It follows that those adolescents with the lowest treatment engagement also have the most undiagnosed and untreated needs^40,41^. This low uptake of services is due to various barriers in help-seeking behaviour for mental health, especially among adolescents^42^. Obstacles include the inability to access mental healthcare, fear of being stigmatised and isolated, lack of awareness, and language or cultural barriers for those with immigrated backgrounds^8^. People need to perceive the need for personal, psychological, emotional or social support before they seek help, and they may not be able to realise or may not be aware when they should seek help or what kind of help is available and how mental health services might improve their well-being^11^. This has been termed the ‘treatment gap’ and has been recognised and is evident worldwide^42^.

According to Andersen’s Behavioural Model of Health Services Utilization^43^, the interplay of perceived health state, health needs, personal health habits, the healthcare system, and the external environment contribute to whether an individual accesses health services. Though, the perception of healthcare needs and utilisation of healthcare services is not the same. Individuals who do not feel a need for services will not avail themselves, and not all individuals who perceive a need will utilise the services available^44^. Perceived need is an essential aspect but not sufficient in considering the need for assistance. Moreover, although higher degrees of impairment and comorbidity are linked with a greater probability that treatment is deemed necessary^44,45^, the mechanisms by which clinical characteristics impact perceived need for treatment and subsequent help-seeking remain unknown^44,46^. Additionally, since some adolescents depend on their parents/caregivers to seek or receive medical treatments, the perceived needs of adolescents might differ from the perceived needs assumed by parents^4^. This difference in perception of mental health care needed maybe due to the lack of understanding about treatment options or causation of mental health problems^4^. Studies show that those who are well informed and diagnosed with a mental disorder have reduced stigmatisation and better awareness of mental health; they are more likely to seek support and services and have better mental health outcomes as they use the services available^47^.

Some public awareness campaigns have successfully bridged the gap between recognising a mental health problem and the perceived need for mental health care and subsequent help-seeking in adults^48,49^. Moreover, past research suggests the importance of assessing initiatives aimed at enhancing awareness of mental health problems^44^. However, limited data have been reported on the perceived need for mental health service access, particularly among adolescents and in the Australian context. For instance, using the same dataset, Schnyder et al.^4^ investigated the patterns of adolescent-parent agreement on perceived need among 13–17-year-olds. While Johnson et al.^26^ reported the perceived need only among those who were diagnosed with any of the four mental disorders (anxiety, depression, ADHD and conduct disorder) in those aged between 4 and 17-year-olds. However, neither of the studies^4,26^ estimated the prevalence of mental health services use by perceived need. Most importantly, none of the studies^4,26^ conducted separate investigations to look at the gap between perceived need for mental health care and actual service use. Moreover, no authors have yet considered both professionally assessed mental disorders and self-reported self-harm and/or suicidality in their papers about mental health care seeking^4,26^. Furthermore, recent studies report that the COVID-19 pandemic has increased the demand for mental health services for adolescents in Australia, it is crucial we better understand who seeks mental health care, ultimately to ensure adolescents have timely access to the available services^50,51^.

This study, therefore, aimed to: (a) estimate the overall prevalence of mental health problems in the study sample (i.e. professionally assessed mental disorders, self-reported self-harm/suicidality and combined), perceived need for care (i.e. what proportion of adolescents think they need mental health services) and service use (i.e. what proportion of adolescents accessed mental health services); (b) assess the association of mental health problems with the perception of a need for mental health service (i.e. determine if those adolescents who have a clear mental health need perceive themselves in need of care); and (c) investigate the relationship of mental health problems with service use using the full sample and then investigating the relationship within two subsamples—adolescents who perceived the need and who did not perceive the need for services.

## Methods

### Data description

The data used in this study was drawn from the Second Australian Child and Adolescent Survey of Mental Health and Wellbeing: the Young Minds Matter (YMM) Survey^52^. The Australian Government Department of Health commissioned the YMM survey, which was carried out by the Telethon Kids Institute, The University of Western Australia, and Roy Morgan Research. A detailed description of the YMM survey has been provided elsewhere^53,54^.

Briefly, YMM was a nationwide cross-sectional study that used a pilot tested Computer-Assisted Personal Interview (CAPI) questionnaire to conduct face-to-face interviews with parents in their homes. At the same time, a previously tested Computer-Assisted Self Interview (CASI) questionnaire was used for adolescents (11–17 years) to gather information related to health-risk behaviours privately at home^53,55^. A stratified, multistage, area-based random sample design (i.e., a child/adolescent was randomly selected if more than one child/adolescent was in the household) was employed. Overall, 6310 parents of children and adolescents aged 4–17 years (55% of eligible households) and 2967 adolescents aged 11–17 years (89% of eligible households) voluntarily participated in the survey between 2013 and 2014^53,54^. The interviews were conducted with representative samples of the nationwide resident population for the children and adolescents aged between 4 and 17 years in Australia. The overall response rate for the parents of children aged 4–17 years was 55%, while the percentage was 89% for the self-reported adolescents aged 11–17 years in Australia^3,53^. However, the YMM survey excluded children from remote regions, homeless children, children in any organisational care, and families where interviews could not be conducted in English^53,54^.

### Ethics

The Human Research Ethics Committees (HREC) of the University of Western Australia and the Australian Government Department of Health (Project 17/2012) approved the YMM research protocol. Later, to access the YMM datasets, our team obtained data access approval from the Australian Data Archive (ADA) Dataverse repository^52,53,55^. Further, the authorship team received ethical approval from the HREC of the University of Southern Queensland for using the YMM datasets to conduct research (HREC Approval No. H16REA205). Written informed consent was obtained for all YMM study participants (i.e., parents and for their children/adolescents). All the investigations were carried out in accordance with appropriate ADA Dataverse guidelines and regulations in using the YMM datasets.

### Measures

Prior studies^44,45^ indicate predisposing factors (e.g. age, gender, education), enabling factors (e.g. geographic location, household income), and health outcome variables (e.g. illnesses, duration, severity) are commonly used to predict the perceived need for and service use among individuals with mental health problems. Thus, based on previous pertinent studies conducted among adolescents^11,26,36^, the variables listed in Table 1 were included in this study to achieve the study objectives.

**Table 1:** List of variables.

#	Variables	Description of variables
**Outcome variables**		
1	Perceived the need	In the YMM survey, a modified version of the Perceived Need for Care Questionnaires (PNCQ)^74^ was used for both parents and self-reporting children (13–17 years) to get information about perceived need for care (i.e., information about services, medication, counselling, and life skills) for any diagnosed mental disorders and/or self-reported self-harm and/or suicidality in the past 12 months prior to the survey. PNCQ is a reliable and validated tool, and previously used for the Australian National Survey of Mental Health and Wellbeing (for adolescents and adults)^4,74^. In this study, for the analytical purpose, we merged parent-reported data and self-reported child data to create a dichotomous variable ‘Perceived the need’ from the Yes/No responses for each of the four categories of perceived need and finally, coded 1 for ‘Yes’ and 0 for ‘No’
2	Service use	Both parents (for their children) and children aged 13–17 years (self-reported) responded about whether the children accessed any of the following services in the previous 12 months—health services (includes general practitioners, psychiatrists, psychologists, community clinics, hospitals), school services, telephone counselling services and online services for any diagnosed mental disorders and/or self-reported self-harm/suicidality with the response options ‘Yes’ and ‘No’ for each category. In this study, during analysis, we merged parent-reported data and self-reported child data, created a binary variable ‘Service use’ for each child in the last 12-months from the Yes/No responses of each service category, and coded 1 for ‘Yes’ and 0 for ‘No’
**Main explanatory variable**		
3	Professionally assessed with mental disorders only	In the YMM survey, initially, seven modules of the Diagnostic Interview Schedule for Children IV (DISC-IV)^75^ were completed by parents for their children, and then, the responses were manually coded either by a qualified psychologist or psychiatrist on the YMM survey team to assess mental disorder/s in children in the past 12-months prior to survey using a structured interview questionnaire. The included mental disorders were major depressive disorder, attention-deficit-hyperactivity-disorder (ADHD), anxiety disorder and conduct disorder ^3^. The responses for each mental disorder included ‘Yes’ and ‘No’. In this study, for analytical purposes, we created a new dichotomous variable ‘Professionally assessed with mental disorders only’ in the past 12-months from Yes/No responses of each mental disorder. We coded 1 for ‘Yes’ when an adolescent was professionally assessed as having at least one of the four mental disorders only and did not self-report self-harm/suicidality
4	Self-reported self-harm/suicidality only	In this study, for the analytical purpose, we created a new binary variable ‘Self-reported self-harm/suicidality only’ in the previous 12-months from Yes/No responses of the following self-reported variables: ‘self-harm’ and ‘suicidality’. We coded 1 for ‘Yes’ when an adolescent self-reported either self-harm/suicidality only and was not professionally assessed as having any mental disorders Self-harm and Suicidality—In the survey, 12–17-year-olds children were directly asked about the experience of self-harm and suicidality in the last 12-months, where all the responses were kept private and not shared with consenting parents. Items measuring self-harm and suicidality were obtained from the validated and tested Standard High School questionnaire of the Youth Risk Behaviour Survey^76^. The following questions measured self-harm and suicidality, respectively: “Have you ever deliberately done something to yourself to cause harm or injury, without intending to end your own life?” and “During the past 12 months, did you ever seriously consider attempting suicide?”^3,53^. All responses were coded 1 for ‘Yes’ and 0 for ‘No’
5	Both	During analysis, from Yes/No responses of ‘Professionally assessed with mental disorders only’ and ‘Self-reported self-harm/suicidality only’ respectively, we created a new variable ‘Both’, for those who were assessed with at least one of the mental disorders and self-reported self-harm/suicidality (coded 1 for ‘Yes’ and 0 for ‘No’)
6	Neither	During analysis, from Yes/No responses of ‘Assessed with mental disorders only’ and ‘Self-reported self-harm/suicidality only’ respectively, we created a new variable ‘Neither’, for those who neither assessed with any mental disorders nor self-reported self-harm/suicidality (coded 1 for ‘Yes’ and 0 for ‘No’)
**Sociodemographic covariates**		
7	Age	Age of the children was categorized into two groups: ‘ > 15 to ≤ 17’ (coded as 1) and ‘13 to ≤ 15’ (coded as 0)
8	Gender	Gender of the children was categorized for both sexes: ‘Girls’ (coded as 1) and ‘Boys’ (coded as 0)
9	Country of birth	Country of birth was categorized into ‘Australian’ (coded as 1), and ‘Overseas’ (coded as 0)
10	Place of residence	According to the Australian Bureau of Statistics (ABS) from the Census of Population and Housing 2016^77^, remoteness areas divide Australia into 5 categories of remoteness based on relative availability of services—major cities, inner regional, outer regional, remote, and very remote. In this study, we created a binary variable ‘Place of residence’ from the responses. ‘Major cities’ was coded as ‘1’, while ‘inner regional’, ‘outer regional’, ‘remote’ and ‘very remote’ were combined to classify as ‘regional/remote’ (coded as 0)
11	Schooling	Whether the adolescent goes to school or not (coded 1 for ‘Yes’ and 0 for ‘No’)
12	Family type	Type of families were categorized on the basis of Australian Bureau of Statistics (ABS)—‘Others’ (coded as 1) and ‘Original’ (coded as 0). Note that ‘Others’ included blended and/or stepparents, foster parents
13	Family functioning	Family functioning was assessed using the items from the McMaster Family Assessment Device (FAD) instrument^55,78^. Then, the YMM research team categorized the responses into poor, fair, good and very good, where very good/good indicating healthy family functioning and fair/poor indicating comparatively unhealthy functioning of the family. In this study, we created a binary variable ‘family functioning’. Those who responded, ‘very good’ or ‘good’ were classified as ‘Good’ (coded as 1), while those who answered, ‘fair’ or ‘poor’ were classified as ‘Poor’ (coded as 0)
14	Parents' Education	Education of the parents was dichotomized into two categories: ‘Bachelor and above’ (coded as 1) and ‘Diploma and below’ (coded as 0)
15	Parents' Employment	Occupation of the parents was dichotomized into two categories: ‘Unemployed’ (coded as 1) and ‘Employed’ (coded as 0)
16	Household income	Equivalised household income is a measure of the economic resources available to each member of a household. It is calculated by using an equivalence factor based on ‘Modified OECD’ equivalence scale and then dividing the income by that equivalence factor^79^. YMM categorized the equivalised household income into low (< $28,000/year)—coded as ‘0’, medium ($28,000–$49,000 per year)—coded as ‘1’, and high (≥ 50,000/year)—coded as ‘2’

### Statistical analysis

**Figure 1:**
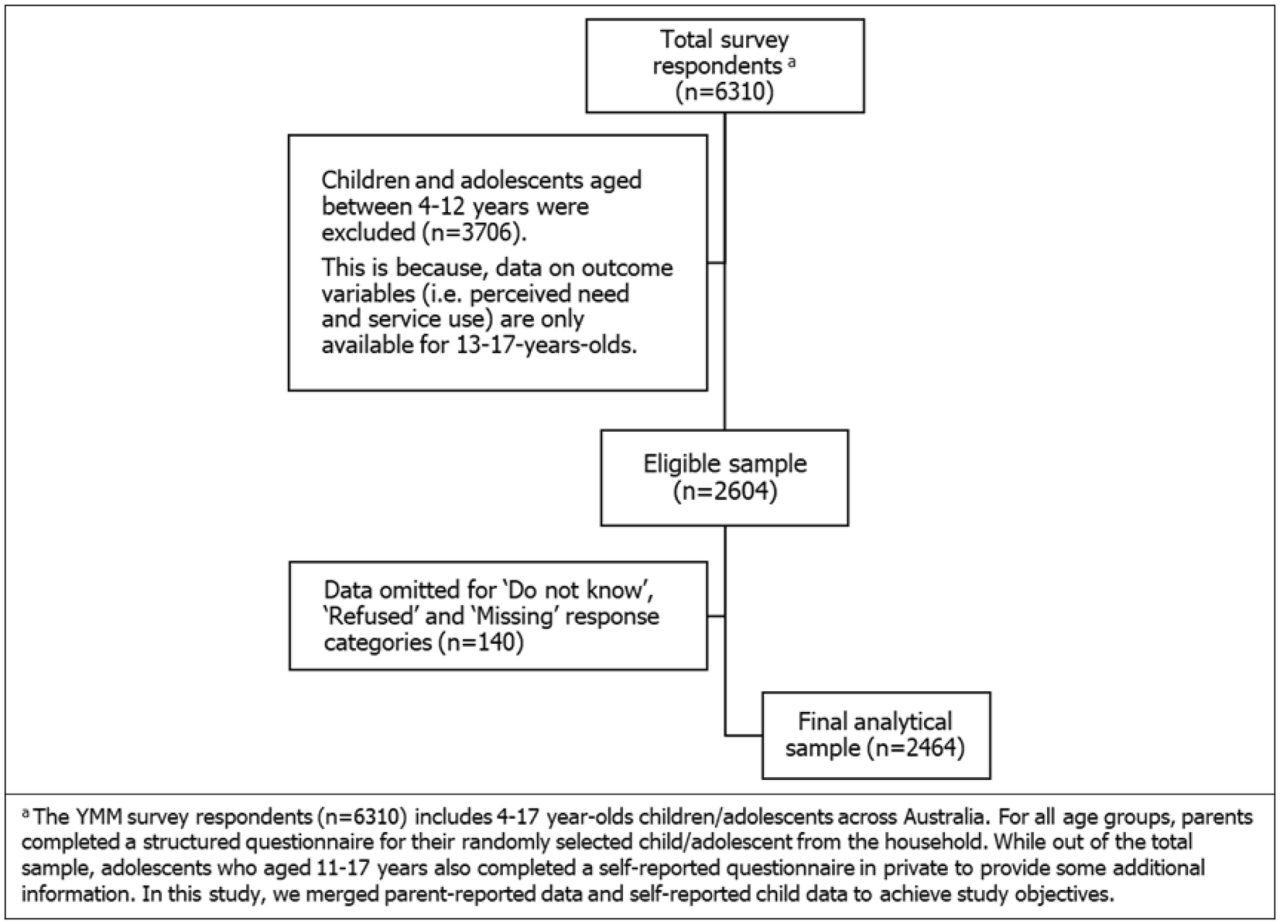
Flow chart for sample selection.

The current study included 2464 adolescents aged 13–17 years of the nationally representative YMM survey. Figure 1 shows the flow chart of the final analytical sample. Respondents included in our study are those who have completed data on the outcome variables (perceived the need for mental health services and actual service use) and the three main explanatory variables (professionally assessed with mental disorders only—major depressive disorder, ADHD, anxiety and conduct disorder; self-reported self-harm and/or suicidality only; and the third group having reported both). The group making up “both” have at least one professionally diagnosed mental disorder and have self-reported self-harm or suicidality. This group is not counted in the other two groups. These variables were obtained from the merged parent-reported data and self-reported child data. During the analysis, the response categories ‘Do not know' and ‘Refused’, and ‘Missing’ were purposively omitted.

All data were weighted in accordance with the YMM survey's cluster sampling methodology, which employed strata and primary sampling units at the country level to ensure that the sample was nationally representative. Descriptive statistics were calculated first to describe sample population characteristics. Then, correlation matrix was estimated to see the strength and direction of relationships between selected variables. Further, Chi-square tests were employed to investigate the bivariate relationships between outcome variables (perceived need and service use) and main explanatory variables (mental health problems). Multivariate logistic regression models were constructed to examine the independent contributions of mental health problems, and sociodemographic covariates on perceived need and service use. Model estimates were reported as adjusted odds ratio (AOR) with 95% confidence interval (CI).

The logistic regression assumptions were evaluated using the McKelvey and Zavoina's R^2^ ^56^ and the Hosmer–Lemeshow Goodness-of-fit test^57^. Lastly, the variance inflation factor (VIF) test was used to find multicollinearity among the independent variables for each model^58^. We used ‘SVYSET’ command for survey design and used Stata software version 14.1 for analysis.

## Results

### Demographic characteristics

For this study sample of 13–17-year-olds (n = 2464), the mean age was 15.4 (SD = 1.38). More than half were (52%) boys, and nearly two-thirds were from major cities (Table 2). The majority (80%) of adolescents attended school, and about 58% were living with their both biological parents. About 68% of adolescents were from families where at least one parent held a diploma and below education. Table 2 also shows more than three-quarters (76%) of adolescents lived in families where their parents were employed and greater than 75% of adolescents were from middle to high income families.

**Table 2:** Demographic characteristics (n = 2464).

Variables	n	%
**Age (Mean = 15.4, SD = 1.38)**		
13 to ≤ 15	1030	41.8
> 15 to 17	1434	58.2
**Gender**		
Boys	1281	52.0
Girls	1183	48.0
**Country of Birth**		
Overseas	353	14.3
Australia	2111	85.7
**Place of residence**		
Regional/Remote	881	35.7
Cities	1583	64.3
**Schooling**		
No	475	19.3
Yes	1989	80.7
**Family type**		
Original	1442	58.5
Others (Blended/Step)	1022	41.5
**Family functioning**		
Poor	463	18.8
Good	2001	81.2
**Parents' Education**		
Diploma and below	1683	68.3
Bachelor and above	781	31.7
**Parents' Employment**		
Unemployed	579	23.5
Employed	1885	76.5
**Household income**		
Low	568	23.1
Medium	1142	46.3
High	754	30.6

### Prevalence of mental health problems

**Figure 2:**
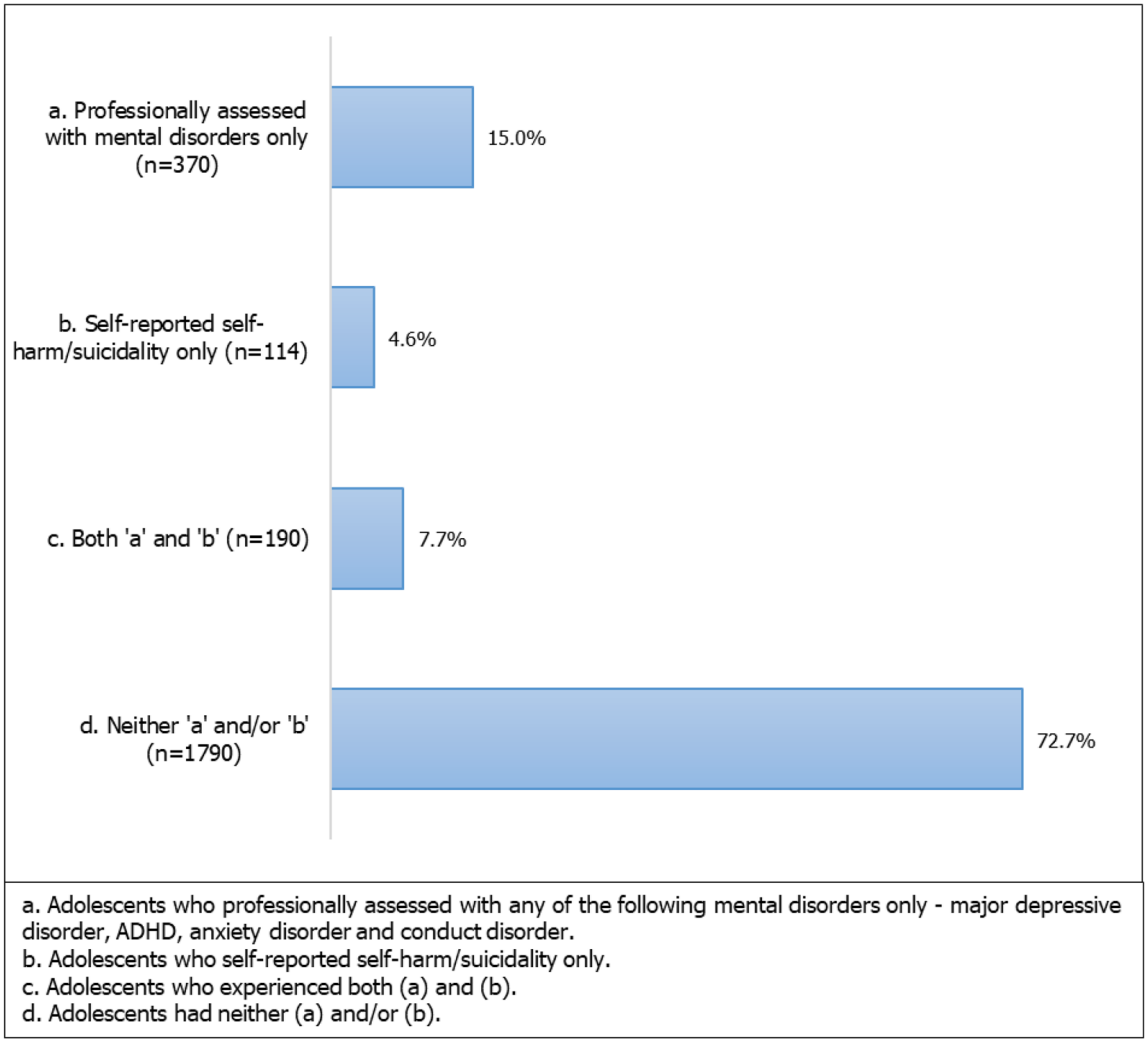
Prevalence of mental health problems.

Figure 2 shows 15.0% (n = 370) of the total sample were professionally assessed with mental disorders only (i.e., any of the following disorders—major depressive disorder, ADHD, anxiety disorder or conduct disorder in the past 12-months). While 4.6% (n = 114) of adolescents self-reported self-harm and/or suicidality only in the previous 12-months. Figure 2 also shows around 7.7% (n = 190) of the sample had reported both, and 72.7% (n = 1790) neither assessed with mental disorders nor self-reported self-harm/suicidality.

### Correlations among study variables

Table 3 portrays the matrix of correlation among the selected study variables. According to the table, mental health problems (professionally assessed with mental disorders only, self-reported self-harm/suicidality only and both) are significantly associated with perceived need for and mental health service use. Perceived need was also linked with service use at 5% significance level. In addition, sociodemographic covariates such as age, gender, country of birth, family type and parental employment were associated with the outcome variables (perceived need for and service use itself), while family functioning and household income was inversely correlated with outcome variables. As expected, among the covariates, parental education, parental employment, and household income were found to be interlinked. Schooling of the adolescents and parental education was also connected.

**Table 3:** Matrix of correlation among study variables.

Variables	1	2	3	4	5	6	7	8	9	10	11	12	13	14	15
1 Age	1.000														
2 Gender	0.024	1.000													
3 Country of birth	−0.023	−0.024	1.000												
4 Place of residence	0.015	0.052	−0.160*	1.000											
5 Schooling	−0.181	0.013	−0.035	0.033	1.000										
6 Family type	0.035	0.017	0.088	−0.018	−0.071	1.000									
7 Family functioning	−0.049	−0.010	−0.010	−0.014	0.036	−0.006	1.000								
8 Parents' Education	0.031	0.007	−0.139	0.111	0.047*	−0.152*	0.026	1.000							
9 Parents' Employment	0.014	0.021	−0.033	0.018	−0.061	0.112	−0.035	−0.130*	1.000						
10 Household income	−0.022	0.005	−0.002	0.094*	0.030	−0.346	0.037	0.288*	−0.305*	1.000					
11 Professionally assessed with mental disorders only	−0.042*	−0.038	0.064*	−0.025	−0.045*	0.114*	−0.071*	−0.039*	0.075*	−0.115*	1.000				
12 Self-reported self-harm/suicidality only	−0.017	0.043*	0.007	−0.025	0.053*	0.015	0.002	0.033	−0.013	−0.010	−0.092*	1.000			
13 Both	0.103*	0.148*	0.027	0.019	0.002	0.084*	−0.059*	−0.030	0.037	−0.036	−0.121*	−0.063*	1.000		
14 Perceived the need	0.067*	0.095*	0.065*	0.023	−0.004	0.128*	0.066*	−0.005	0.057*	−0.070*	0.249*	0.135*	0.280*	1.000	
15 Service use	0.022	0.107*	0.072*	0.000	−0.006	0.157*	−0.041*	−0.031	0.102*	−0.118*	0.268*	0.072*	0.265*	0.546*	1.000

Total observations, n = 2464, *p < 0.05 considered significant.

### Associations of mental health problems with perceived need and service use

Table 4 depicts that 47.4 and 27.5% of the total analytical sample perceived the need for mental health services and used these services, respectively. In addition, about 53% of adolescents who perceived the need for mental health services, used mental health services; while the percentage of using services was around 4% for those who did not perceive the need for mental health problems.

**Table 4:** Association of mental health problems with perceived need and service use (Bivariate analysis).

	Perceived the need			Service use			Service use among those who perceived the need			Service use among those who did not perceive the need		
	No	Yes	*p*-value	No	Yes	*p*-value	No	Yes	*p*-value	No	Yes	*p*-value
Total	1297 (52.6)	1167 (47.4)		1787 (72.5)	677 (27.5)		546 (46.8)	621 (53.2)		1241 (95.7)	56 (4.3)	
**Professionally assessed with mental disorders only**			< 0.001			< 0.001			< 0.001			0.198
No	1212 (57.9)	882 (42.1)		1624 (77.6)	470 (22.5)		462 (52.4)	420 (47.6)		1162 (95.9)	50 (4.1)	
Yes	85 (22.9)	285 (77.1)		163 (44.1)	207 (55.9)		84 (29.5)	201 (70.5)		79 (92.9)	6 (7.1)	
**Self-reported self-harm/suicidality only**			< 0.001			< 0.001			0.458			0.004
No	1272 (54.1)	1078 (45.9)		1721 (73.2)	629 (26.8)		501 (46.5)	577 (53.5)		1220 (95.9)	52 (4.1)	
Yes	25 (21.9)	89 (78.1)		66 (57.9)	48 (42.1)		45 (50.6)	44 (49.4)		21 (84.0)	4 (16.0)	
**Both**			< 0.001			< 0.001			< 0.001			0.253
No	1289 (56.7)	985 (43.3)		1727 (75.9)	547 (24.1)		493 (50.1)	492 (49.9)		1234 (95.7)	55 (4.3)	
Yes	8 (4.2)	182 (95.8)		60 (31.6)	130 (68.4)		53 (29.1)	129 (70.9)		7 (87.5)	1 (12.5)	

The bivariate analysis in Table 4 shows that about 77.1, 78.1 and 95.8% of adolescents who were professionally assessed as having mental disorders only, who self-reported self-harm/suicidality only and who reported both, respectively, perceived the need for care in the previous 12-months (*p* < 0.001 for all). In addition, those professionally assessed with mental disorders only, self-reported self-harm/suicidality only and both were found to be associated with any service use in the past 12-months at a 1% significance level. Table 4 also illustrates that among those who perceived the need, approximately 70% (*p* < 0.001), 49% (*p* = 0.458) and 71% (*p* < 0.001) with professionally assessed mental disorders only, self-reported self-harm/suicidality only and both used any services in the past 12-months, respectively. That means a significant proportion of adolescents perceived the need but did not use any mental health services in the last 12-months, indicating a treatment gap. Surprisingly, among those adolescents who did not perceive the need, self-reported self-harm/suicidality only was found to be significantly associated with service use (*p* < 0.01).

### Odds of perceived need and service use

The results of the logistic regression models that investigated the association of mental health problems (professionally assessed with mental disorders only, self-reported self-harm/suicidality only and both) with perceived need and service use among adolescents aged 13–17 are summarized in Table 5. All models were adjusted for potential sociodemographic covariates.

**Table 5:** Logistic regression models predicting perceived need and service use.

Variables	Model I		Model II		Model III		Model IV	
	Perceived the need		Service use		Service use among those who perceived the need		Service use among those who did not perceive the need	
	aOR (95% CI)	VIF	aOR (95% CI)	VIF	aOR (95% CI)	VIF	aOR (95% CI)	VIF
Age (ref. 13 to ≤ 15)		1.08		1.12		1.12		1.22
> 15 to 17	1.28** (1.06, 1.54)		1.02 (0.83, 1.26)		0.99 (0.76, 1.28)		0.52* (0.29, 0.90)	
Gender (ref. Boys)		1.05		1.08		1.09		1.24
Girls	1.27** (1.06, 1.52)		1.47*** (1.20, 1.80)		1.42** (1.11, 1.83)		1.39 (0.80, 2.41)	
Country of Birth (ref. Overseas)		1.06		1.05		1.05		1.23
Australia	1.37* (1.05, 1.79)		1.47* (1.07, 2.01)		1.54* (1.04, 2.28)		0.65 (0.33, 1.29)	
Place of residence (ref. Rural/Remote)		1.05		1.03		1.03		1.14
Major cities	1.17 (0.97, 1.42)		1.04 (0.85, 1.28)		0.92 (0.71, 1.20)		1.00 (0.56, 1.81)	
Schooling (ref. No)		1.07		1.08		1.07		1.42
Yes	1.08 (0.85, 1.36)		1.07 (0.83, 1.39)		0.99 (0.72, 1.36)		2.06 (0.85, 5.00)	
Family type (ref. Original)		1.18		1.16		1.17		1.35
Others (Blended/Step)	1.37** (1.13, 1.67)		1.51*** (1.22, 1.87)		1.29 (0.99, 1.68)		1.81* (1.00, 3.26)	
Family functioning (ref. Fair/Poor)		1.03		1.05		1.06		1.08
Good/Very good	0.85 (0.67, 1.07)		1.00 (0.77, 1.28)		1.13 (0.84, 1.53)		0.91 (0.44, 1.86)	
Parents' Education (ref. Diploma and below)		1.15		1.16		1.16		1.40
Bachelor and above	1.23* (1.01, 1.51)		1.13 (0.90, 1.42)		0.96 (0.74, 1.28)		1.23 (0.66, 2.28)	
Parents' Employment (ref. Employed)		1.15		1.15		1.18		1.11
Unemployed	1.14 (0.91, 1.43)		1.38** (1.08, 1.76)		1.27 (0.93, 1.72)		2.18* (1.17, 4.06)	
Household income quintile (ref. Low)		1.35		1.38		1.41		1.55
Medium	1.02 (0.80, 1.31)		0.85 (0.65, 1.11)		0.78 (0.55, 1.09)		0.82 (0.40, 1.68)	
High	0.95 (0.72, 1.27)		0.76 (0.56, 1.05)		0.70 (0.48, 1.03)		0.91 (0.40, 2.09)	
Professionally assessed with mental disorders only (ref. No)		1.16		1.23		1.28		1.15
Yes	6.22*** (4.76, 8.12)		5.96*** (4.65, 7.64)		3.27*** (2.39, 4.47)		1.75 (0.71, 4.33)	
Self-reported self-harm/suicidality only (ref. No)		1.07		1.09		1.11		1.11
Yes	6.65*** (4.20, 10.52)		3.47*** (2.32, 5.17)		1.40 (0.89, 2.20)		4.49* (1.43, 14.03)	
Both (ref. No)		1.16		1.25		1.29		1.19
Yes	37.35*** (18.21, 76.58)		9.31*** (6.62, 13.09)		3.15*** (2.18, 4.57)		3.28 (0.36, 29.52)	
**Model performance tests**								
McKelvey & Zavoina's R^2^	0.311		0.228		0.136		0.146	
Hosmer–Lemeshow statistic (p-value)	9.81 (0.831)		5.09 (0.991)		12.37 (0.650)		544.51 (0.311)	
Mean VIF (Max)	1.12 (1.35)		1.14 (1.38)		1.16 (1.41)		1.25 (1.55)	

Level of significance: ***p < 0.001, **p < 0.01, *p < 0.05.

*aOR*  Adjusted odds ratio, *CI*  Confidence interval; *VIF*  Variance inflation factor.

Model I in Table 5 shows that adolescents who were professionally assessed with mental disorders only, self-reported self-harm/suicidality only and had reported both were respectively 6.22 times (95% CI: 4.76–8.12), 6.65 times (95% CI: 4.20–10.52) and 37.35 times (95% CI: 18.21–76.58) more likely to perceive need for care compared to those who did not experience any mental health problems. Similarly, Model II indicates that those who were professionally assessed with mental disorders only (aOR 5.96, 95% CI: 4.65–7.64), self-reported self-harm/suicidality only (aOR 3.47, 95% CI: 2.32–5.17) and both (aOR 9.31, 95% CI: 6.62–13.09) were more likely to use any services compared to their counterparts. While Model III revealed that adolescents who were professionally assessed with mental disorders only (aOR 3.27, 95% CI: 2.39–4.47) and those who reported both (aOR 3.15, 95% CI: 2.18–4.57) were significantly more likely to use services among those who perceived the need for care compared to their counterparts. Further, Table 5 indicates the significant association between self-reported self-harm/suicidality only and service use among those who did not perceive the need for care in Model IV (aOR: 4.49, 95% CI: 1.43–14.03).

In addition, the findings revealed that girls, those who were born in Australia, those not living with their biological parents and having parents who have completed bachelor or above education were more likely to perceive the need (Model I) and use services (Model II, except for parental education) for mental health care compared to their counterparts (*p* < 0.05 for all). Model I also found that older adolescents (15–17 years) were more likely to perceive the need for care in comparison to younger aged adolescents. Model II found adolescents with unemployed parents were more likely to use services than those from employed parents. While in Model III, only gender and country of birth were found to be associated with service use among those who perceived the need for care compared to their counterparts (*p* < 0.05 for all). In addition, family type and parental employment were found to be significantly associated with service use among those who did not perceive need for mental health problems in Model IV (*p* < 0.05) in Table 5.

### Model performance results

Lastly, Table 5 depicts the results of several statistical diagnostic tests used to verify the precision of estimates arising from the regression models. For instance, McKelvey and Zavoina R^2^ values were smaller than one, and the Hosmer–Lemeshow Goodness-of-fit tests reveal no significant discrepancy between the models and the observed data (*p* > 0.05), indicating that the models were well-fitted. Additionally, the VIF with a mean of 1.20 for each model suggested no indication of multicollinearity among predictor variables.

## Discussion

This study expands our understanding from previous research by providing further evidence on the perceived need for and mental health service use for 13–17-year-olds adolescents who had mental health problems in the past 12-months; using definition approach recommended by WHO (i.e., professionally assessed with mental disorders only—depression, anxiety, ADHD, conduct disorder; self-reported self-harm/suicidality only; and both). Our study used data from the latest nationally representative, mental health survey among children and adolescents in Australia—Young Minds Matter^53^. The findings of our study substantiated and extended beyond those from previous studies conducted in Australia by Schnyder et al.^4^ and Johnson et al.^26^. In particular, our findings demonstrate that adolescents who were professionally assessed with mental disorders only, self-reported self-harm/suicidality only or reported both were significantly more likely to perceive the need for care and engage in actual service use. Importantly and uniquely, our research has also found that a significant proportion of adolescents who perceived the need for care were not using mental health services, and self-reported self-harm/suicidality only was significantly associated with service use among those adolescents who responded that they did not perceive the need for mental health services.

Our study revealed that almost half of the sample (47.4%) perceived the need for care, which was consistent to that reported by Schnyder et al.^4^ and Johnson et al.^26^. An earlier study carried out among adults in multiple developed countries also reported similar prevalence, primarily determined by psychiatric morbidity^44^. Further, we observed in our analyses that only around a quarter of the sample (27.5%) used any mental health services, which is relatively low compared to past studies, where previous study estimates vary between 30 and 40%^46,59^.

Furthermore, our study found the adolescents who were professionally assessed with mental disorders only and reported both a professionally assessed mental health condition and self-harm/suicidality were more likely to perceive the need for and seek mental health services than those who had not been professionally assessed with any disorder. Adolescents who experience significant burden associated with a mental disorder (e.g., inattention, worrying, sadness, social isolation, interruption in work, lower educational achievement, financial burden, reduced quality of life)^60–62^, may motivate individuals to seek mental health services to help their diagnosis in order to improve the poor mental health conditions. In addition, our study revealed that adolescents who self-reported self-harm and/or suicidality only were more likely to perceive the need for care and seek mental health services (statistically significant but low in effect compared to perceived need). While self-reported self-harm/suicidality only was not significantly associated with service use among those who perceived the need for these services. Possible barriers to accessing services among those who perceive the need to seek these services commonly include worries about being stigmatized, fear of embarrassment, lack of knowledge and understanding of the help-seeking process and importantly, a desire to solve problems on their own without involving others (eg. parents)^62,63^. Interestingly, our study found that self-reported self-harm/suicidality only was associated with higher likelihood of using services among those adolescents who did not perceive the need for care. This may be because adolescents did not perceive the need for care earlier and potentially accessed the available service on an emergency basis only after a crisis incident (self-harm/suicidality). Previous studies have found that adolescents with self-harming/suicidal behaviour are more likely to seek help from peers, counsellors and health professionals but are less likely to seek help from parents, siblings or relatives^64,65^.

Further, our study highlights a significant disparity between the perceived need for mental health services and actual service use. Overall, about half of the adolescents (47%) did not seek mental health services although they perceived the need for care. This gap is consistent with earlier studies, where contextual factors and family influences (parents not realising their adolescent needs help) were reported as key^65,66^. This conflict between what a parent thinks about their adolescent’s mental health versus how the adolescent is doing indicates a major mismatch regarding adolescents’ mental health. A previous study pointed to a poor adolescent-parent agreement regarding adolescent mental health, and this was linked to inadequate parental knowledge about adolescent’s feelings^36^. Based on our findings and earlier evidence, this suggests that the parent plays a major role in deciding whether their adolescent accesses mental health services and parents act on what they observe, know about, or feel is important, but the observations made by the parent may not adequately reflect how the adolescent is doing in terms of their mental well-being. This knowledge highlights areas for improvement. Improvements might include assisting parent-adolescent communication of mental health needs, further bolstering services and processes that enable adolescents to seek care independent of the parent, education for parents regarding the range of mental health needs and what health services can do to help. Continuing improvements in mental health services for adolescents might consider that Australian adolescents with self-harm/suicidality are more likely to use online and in-person health services such as GP, psychologists, paediatricians, hospitals, private clinics, emergency departments and counsellors/family therapists and are less likely to use telephone services^27,35,36^.

Moreover, our study found that adolescents born overseas were less likely to perceive need for mental health care and seek services compared to those who were born in Australia. This may be due to cultural barriers in terms of perceived social stigma regarding mental health conditions, embarrassment and mental health illiteracy^63,67,68^. Prior studies have shown that girls are more likely to seek mental health services and are more likely to perceive the need for services than boys^45^; our study supports this statement. Additionally, our study found adolescents who live with blended/stepparents were more likely to perceive need and use mental health services, which is consistent with the findings from a previous study that reported family circumstances are significantly associated with perceived mental health needs and service access^69^. Furthermore, the findings of our study suggested that highly educated parents increase the likelihood of perceived need for care among adolescents. This may be because parents with higher education have better knowledge of mental health problems than parents with comparatively lower educational level^63^. In addition, our study estimated that 23.5% of parents were unemployed, this is because mother was primarily included as the parent and/or caregiver by the YMM survey. Latest reports on labour force status of families by the Australian Bureau of Statistics estimated that 74% of mothers with adolescents in couple families are employed^70^.

Using the latest national representative survey data is one of the major strengths of our study since it has previously been concluded that the YMM survey broadly represents the Australian adolescent population on major demographic characteristics^53^. Further, the YMM survey included professionally assessed mental disorders and standardised items for self-reported mental health conditions (self-harm/suicidality) for children and adolescents as described by the World Health Organization (WHO)^7^. In addition, having merged report (parent-reported data and self-reported data) on perceived need and service use is a strength of the study. However, our study has several limitations. First, the cross-sectional nature of the YMM survey, limits our ability to understand temporal causality between explanatory and outcome variables. Second, the YMM survey was conducted only among non-Indigenous 4–17 years adolescents in Australia; thus, the results may not be generalized for Australian adults and Indigenous adolescents. Third, information regarding self-harm and suicidality were self-reported and hence, measurement error and recall and/or response bias may be affecting the data^71^. Although previous studies validated self-reporting as the most plausible method for health risk behaviours among children and adolescents^72,73^. Fourth, important variables such as substance use disorder, eating disorder and post-traumatic stress disorder were not available in the YMM survey dataset, this limits the findings of the study. Lastly, the survey lacked an evaluation of whether the service use improved adolescents' mental health, their school performance or social functioning.

## Conclusions

This study found that a significant proportion of adolescents who perceived the need for care did not use mental health services and this was particularly stark for adolescent’s experiencing self-harm and/or suicidality. The mental health needs of adolescents were already high in Australia and globally, and COVID-19 has exacerbated this. While funds are being dedicated to increase mental health services in Australia, it will be crucial to act on findings such as those in this paper, which demonstrate a wide treatment gap exists for adolescents with mental health needs who do not seek care.
